# Supplementary material for: Convergence in voice fundamental frequency during synchronous speech
Source: PLoS One. 2021 Oct 21;16(10):e0258747. doi: 10.1371/journal.pone.0258747 (PMC8530294; doi:10.1371/journal.pone.0258747)
Supplement: S1 Table — Sentences taken from the Harvard IEEE corpus of sentences used in Experiments 1 and 2. (PDF) [file pone.0258747.s003.pdf]

**Sentence stimuli**	
**Solo reading and synchronous speech task Experiment 1**	
The birch canoe slid on the smooth planks.	Two blue fish swam in the tank.
Glue the sheet to the dark blue background.	Her purse was full of useless trash.
It's easy to tell the depth of a well.	The colt reared and threw the tall rider.
These days a chicken leg is a rare dish.	It snowed, rained, and hailed the same morning.
Rice is often served in round bowls.	Read verse out loud for pleasure.
The juice of lemons makes fine punch.	The frosty air passed through the coat.
The box was thrown beside the parked truck.	The crooked maze failed to fool the mouse.
The hogs were fed chopped corn and garbage.	Adding fast leads to wrong sums.
Four hours of steady work faced us.	The show was a flop from the very start.
A large size in stockings is hard to sell.	A saw is a tool used for making boards.
The boy was there when the sun rose.	The wagon moved on well oiled wheels.
A rod is used to catch pink salmon.	March the soldiers past the next hill.
The source of the huge river is the clear spring.	A cup of sugar makes sweet fudge.
Kick the ball straight and follow through.	Place a rosebush near the porch steps.
Help the woman get back to her feet.	Both lost their lives in the raging storm.
A pot of tea helps to pass the evening.	The slush lay deep along the street.
Smoky fires lack flame and heat.	A wisp of cloud hung in the blue air.
The soft cushion broke the man's fall.	A pound of sugar costs more than eggs.
The salt breeze came across from the sea.	The fin was sharp and cut the clear water.
The girl at the booth sold fifty bonds.	The play seems dull and quite stupid.
The small pup gnawed a hole in the sock.	Bail the boat to stop it from sinking.
The fish twisted and turned on the bent hook.	The term ended in late june that year.
Press the pants and sew a button on the vest.	A Tusk is used to make costly gifts.
The swan dive was far short of perfect.	Ten pins were set in order.
The beauty of the view stunned the young boy.	The bill was paid every third week.

**Practice stimuli for synchronous speech task Experiment 1**
Use a pencil to write the first draft.
The two met while playing on the sand.
The ink stain dried on the finished page.
The walled town was seized without a fight.
The hat brim was wide and too droopy.
**Solo reading and synchronous speech task Experiment 2**
The bill was paid every third week.
The juice of lemons makes fine punch.
The wagon moved on well oiled wheels.

Sentences were taken from the Harvard IEEE corpus of sentences:

IEEE Subcommittee on Subjective Measurements. (1969). IEEE Recommended Practice for Speech Quality Measurements. *IEEE Transactions on Audio and Electroacoustics*, *17*(3), 227–246.
