# Supplementary material for: Convergence in voice fundamental frequency during synchronous speech
Source: PLoS One. 2021 Oct 21;16(10):e0258747. doi: 10.1371/journal.pone.0258747 (PMC8530294; doi:10.1371/journal.pone.0258747)
Supplement: Attachment — Submitted filename: PLoSSynchSpeech.pdf [file pone.0258747.s004.pdf]

This review will not be anonymous.

The manuscript reports a series of experiments examining F0 changes during synchronous speaking. There has been little work reported on changes to F0 under these conditions, so there is a gap here to be filled. The work is all carried out under the onerous conditions of the pandemic, which has constrained what can be done greatly, and it is to be hoped that this is regarded as preliminary to a less constrained study. Overall, the work reported here should be a valuable contribution to a growing literature that can guide further inquiry. As the manuscript stands, there are issues with how results are reported that require substantial revision. I am of the view that this paper should be much much shorter, with less statistical analysis. Analysis should make clear the very simple effects found, and should avoid complicating matters with wrongly used technical corrections and discriminations.

One obvious limitation of the constraints is the uncontrolled nature of the recordings which are done by subjects using their own computers and microphones. This is addressed sensibly as far as possible, and the inclusion of the Higgins Pitch test and the vigilance check go some way to ameliorating the limitation. It seems to this reviewer that usable F0 data might be obtained in this fashion.

A second limitation is the use of pre-recorded voices with which subjects are asked to align. The difference between coordination with a recording, and reciprocal coupling with a live voice is an extremely important one (as we found in Jasmin et al, 2015, and elsewhere). It is important for many reasons, which the authors are aware of. The motivating interest in choral speech support for stutterers makes this somewhat less of an issue, but it greatly reduces the likelihood that our understanding of joint speech production will be furthered by this study. The use of recordings, rather than live co-speakers, has the additional effect of supporting interpretation in terms of individualistic representational models, as here, rather than dynamical embodied models, as becomes more attractive when the intersubjective situation of co-speaking is employed. This thrust towards one kind of model rather than another is not acknowledged. More on those models later.

Which leads naturally to the term "choral speech". Having specialized in all forms of collective speaking over many years, I have tried to encourage others to adopt a coherent terminology. The original work on stuttering did indeed use the term "choral speech". This term otherwise normally refers to the performative group recitation of poems and the like performed, often competitively, by children in South East Asia and in Ireland (at least). The term Synchronous Speech, I have argued ought to be reserved for the kind of laboratory experiment done here, while the umbrella term Joint Speech has the useful function of covering all occasions in which multiple people utter the same sounds at the same time. I cannot enforce terminological uniformity, but I can plead with my colleagues that these considerations should encourage us to use terms in a manner that is well aligned. The original work on stuttering long predates these additional concerns, and it is to be hoped that the field will become more consistent over time.

There is little prior work on F0 and synchronous speech. The authors note Cummins (2000), which noted that pitch range is reduced in synchronous speaking conditions. They have not noted Cummins (2004) which documented lower intonational variability (peaks and valleys) under synchronous speaking conditions. Understandably absent is Wang & Cummins (2003) which is just a published abstract, but it examined details of intonation contours in a tonal language, which brings me to a substantial point: the only variable employed here is the median F0 over a sentence length utterance. This variable is suited to a coarse examinination of phonetic convergence, as here, but it omits a lot of detail that is possibly also of interest, including range, variability, and slope. I would venture to suggest that a more fulsome examination of F0 under these conditions ought to employ

more detailed measurements, if they are to be of use in adjudicating the claims of competing models.

There are 4 experiments. I will go through them in turn.

Experiment 1: Here, convergence towards the recorded target is observed both downwards and upwards. That result was to be expected and is nice to see. I had great difficulty with the manner in which the results are reported, and I suggest that a substantial rewrite is needed if the important features observed are to be brought forth, and confusing details relegated to the background.

A pragmatic choice to use only female speakers was made. This seems fine and justifiable. However it does not remove the messiness of human physiology from the equation and propel us into a metric space in which meaningful distances can be measured. Each speaker has her own larynx, her own idiosyncrasies of movement, and can be assumed, in a first approximation, to have something like a center, or mid-point, from which excursions upwards and downwards will require speaker-specific effort. It cannot be assumed that each speaker is free to raise or lower their F0 to an arbitrary extent, or that different speakers have similarly "elastic" pitches. In the ensuing analysis, the authors assume that the median F0 obtained in the solo speaking task is, for each speaker, a kind of "baseline." I'm afraid that term gave me the greatest difficulty, and I will try to explain why. (It took very many readings to discover what is being reported here... I may still be misinterpreting things.)

These speakers are now given the task of speaking along with recordings that have been prepared to have relatively high or low median F0. The experimental result obtained is evidence that when speaking in synchrony there is a shift in median F0 upwards or downwards, depending on condition. That is the result, but it is hidden in a barrage of non-results that I will now look at.

The t-tests (p. 18, lines 412ff) document this shift. They are done on a per-subject basis, which is entirely appropriate.

The difficulties start on p. 19, line 427. "At the group level, average baseline F0 (at solo reading) did not differ between high (M = 193.86, SD = 25.28) and low (M = 198.21, SD = 26.07) F0 conditions (t(17.98) = -0.379, p = .709)." This took me forever to decode. If I understand correctly, this is a comparison of the solo speech from two groups, given the between subjects design. Its purpose is to verify that the between subjects design is reasonably justifiable. Large differences here might lead one to question the equivalence between groups, threatening the experimental design.

However the reporting is done in terms of the "high" and "low" conditions. These are utterly irrelevant and misleading. It is true that each group will go on to speak with recordings that are high or low, but the analysis here is of the solo recordings only. The experimentally manipulated recordings play no role. This should be reported as a check of equivalence between groups, prior to exposure to experimental contrasts. It should not be framed in terms of those experimental contrasts. The manner in which this is reported destabilized the notion of a baseline for me and led to hours of alternative consideration as to what baseline might be meant. I strongly suggest not using that term, but if it is used, it should not be subjected to this kind of problematic confusion with irrelevant factors (High and Low are irrelevant here. It is simple inter-group comparability that is being tested.)

A second problematic report follows, on Line 433: "However, distance in F0 from the accompanist voice at baseline did differ between conditions." This still makes my head spin and makes the "baseline" more mysterious yet. The syntax alone does not make it clear who owns the baseline, and it suggests that "the accompanist voice at baseline" is a thing. It is not a thing, and I lost more time over this. My best interpretation of this assertion is that the degree of convergence observed in each

group was not identical, and this rather simple result is augmented by the much more problematic claim that there was "a greater F0 distance at baseline in the high condition (M = 70.56, SD = 25.13) compared to the low condition." This is not only confusing, but confused. The distances that are summoned to warrant the terms "greater" and its assumed "lesser" for the low condition, are conjured up by the experimenter. They are not observations, but design decisions. The same experiment could have been run with target recordings that had been manipulated to have higher or lower values. If we leave the high target the same, but drop the low target by 50 Hz, the result will be different, not because of anything the subjects did, but because we designed things that way. Again, I may be misunderstanding things, but if I am, something is still clearly amiss that such misunderstanding after diligent effort should be possible.

My interpretation is supported by the ensuing claim (line 436...): "This indicates that participants in the high condition started the task with an F0 that was further away from their corresponding accompanist's F0 compared to those in the low condition with their respective accompanist's F0." Is this not obviously just the arbitrary values the authors chose to place the F0 of the accompanist at? The phrase "started the task" confused me further. There is no analysis here of a temporal progression or time series, which is what those words suggest. Here the authors seem to be acknowledging that "well, we just put this here" and reporting that as a result. I understand that the "baseline" is different for each participant, and so they might differ in where their zero point lies between the two extremes, but the extremes are set by the experimenter.

Then the first hypothesis is stated: "Hypothesis 1: Change in produced F0 values during choral speech will be significantly different between high and low F0 conditions". When a hypothesis is stated, it must first be motivated and argued for. This has not happened, and this appears out of the blue. Furthermore, upon examination, it has the same problem just mentioned. That there might be convergence towards the HI and LO targets is clear, agreed, and found. But to express this as a magnitude that can be quantitatively compared across conditions raises two substantial problems. 1: as noted, the targets have been somewhat arbitrarily set by the experimenters, so any asymmetry in degree of convergence is at least partly built in, not found. 2: The difference alluded to in the hypothesis is metric. The voice is not an abstract thing. Each speaker will have personal limits on how far, and fast, they can modulate their voice. If we were to (wrongly) suggest that subjects are actually striving to reach the value of the accompanist, it would surely be clear that this represents a different challenge for each speaker, depending on their capacity for vocal modulation. Of course, many experimental designs specifically set out to wash out such inter-participant differences by averaging, but that is to treat the voice as if it were itself abstract. The authors chose not to include men, for their inclusion would make it obvious that averaging is inappropriate. The problem does not go away with reduction to a single sex.

While the latter problem may be unavoidable, it matters, and the hypothesis that is conjured up out of the blue needs to first consider such factors, and to put forward arguments relating not only to direction, but to magnitude, which is not straightforwardly measurable. The LMM analysis that follows is intended to make manifest metric differences, and thus to interpret magnitudes. But there are no objective magnitudes possible when the experimenters design not only the axis of measurement, but its underlying topology. Consider an analogy. I want to see how high a bunch of students will jump, so I hold out a bar and encourage them to jump over it. The bar is higher than they can jump, but they valiantly try. Another teacher does the same experiment but makes the bar much higher. Students faced with the higher target try harder, and their jumps have a different distribution than my students. The analogy is not great, but perhaps it can illustrate how any magnitudes measured under such circumstances depend in a serious way on the choices of the experimenter beforehand.

Despite all these issues, the finding seems perfectly clear and could be demonstrated in a simpler manner and with less possibility for confusion. "Therefore, as predicted, F0 change from baseline was positive in the high condition, but negative in the low condition. This suggests that the acoustic properties of the accompanist's voice (specifically, F0) did affect the nature of the change in the participants' voices at choral speech." This is clear, correct, and of interest. It does not invoke magnitudes.

Hypothesis 2 is then provided: "Hypothesis 2: F0 distance will be lower at choral speech compared to solo reading in both high and low conditions"

Once more, this hypothesis is created out of air. There has been no argument to motivate or justify it.

I find it uninterpretable. What distance is meant? The figures do not have any captions, despite the problematic practice of the journal of placing them at the end of the manuscript. When that is done (sigh) there should at least be a list of figure captions. The abbreviations SR and CS I have reverse engineered to deduce they mean Solo Reading and Choral Speech. Given that reading is not at stake here, the SR abbreviation (if I am guessing correctly) is misleading. At the bottom left of Fig 3 the two words "phase" and "condition" occur. Do they belong together? "Phase" is a word with a lot of meanings. What could it possible mean here? And most importantly of all, the Y-axis is F0 distance, but nowhere does it say distance between what and what.

The hypothesis that came from nowhere is expanded on thus (p. 20, line 471...): "To test whether the distance between participant and accompanist F0 values was smaller at choral speech compared to solo reading, an LMM analysis was used on F0 distance values." This is unclear. The accompanist does not provide solo readings (again, "reading" is misleading, when speaking is at issue). If I try to rephrase this, I think it means that median F0 values measured in the choral speech condition are displaced towards the F0 values of the manipulated recordings. I did not use the word "distance" in that reformulation. Perhaps I have misunderstood, but that seems to me to be the sense.

I have no desire or skill to get into the weeds of debating the specific statistical tests used, but they should be testing something meaningful, and something that we have been primed by argument to expect, and to be able to interpret. An unmotivated or explained hypothesis followed by statistics quickly causes the reader to get lost, and to fail to see what the result obtained is. The LMM model is described thus: "The interaction effect in this model indicated that the difference between F0 distance at baseline and choral speech was greater (more negative) in the high condition compared to the low condition." This statistical test is concerned with the estimation of magnitudes, and as discussed above, the metric that such measurement relies on is not warranted, but arises as a function of the contingent targets set by the experimenters combined with the individual physiology of the participants considered individually. The ensuing dive into Bonferroni correction and whatnot does nothing but obscure the perfectly simple result. An analysis like this achieves nothing, but casts a magical air of scientism over something which is nothing but a simple effect. Interpreting quantitative inferential statistics is difficult at the best of times, but it demands that the scales, magnitudes, and effects be sensible, well motivated, and transparent. That is not the case here.

Discussion of Experiment 1: "Overall, experiment 1 found evidence of significant convergence in F0 to an accompanist voice during choral speech, both when convergence required a lowering of F0 (low F0 accompanist condition) and when it required a raising of F0 (high F0 accompanist condition)." This seems fine, secure, and is interpretable. But "However, our analysis found that convergence in F0 was greater in the high F0 condition compared to the low F0 condition." This is uninterpretable and adds nothing. See all my points about measurement, metrics, and physiology.

The authors see some of what I am trying to point to: "However, a flaw in the current experimental design was that the high and low conditions did not start out with an equivalent distance between participant and accompanist F0s at baseline, with a greater distance at baseline in the high F0 compared to the low F0 condition." Yes, but it is not really a flaw. The experiment was designed thus, and there is no way to fix it. Even if you had equal Hz values between baseline and HI and LO, respectively, that numerical equivalence is projected onto a space of flesh, spit and muscle. Equal Hertz is equality within a representational scheme only. There is no fix, any more than one can aspire to collating unbiased data over a non-ergodic, ill-defined, and unknowable population. This certainly has nothing to do with differences between Americans and Brits.

The discussion sets up Experiment 2, more or less in line with my analogy above, by employing a target even more remote from the subject's baseline. That is fine, as far as it goes, but no quantitative conclusions can be drawn from such a modified design. One can ask whether using more remote "targets" (they are not targets, of course) produces a noticeable effect. One can ask nothing sensible about magnitudes, because one does not have command of the underlying space being represented, which is built of flesh, volition, ambition, perceived difficulty, and such intangible quantities.

The comparisons done in Experiment 2 are uninterpretable. The experimenters have no command of the underlying reality, and are forced to compare the HI condition of Expt 1 with the XLO condition of Expt 2. This once more makes the larynx into an abstract thing, to whom up and down are mere directions. Distance from baseline towards a high target are being compared numerically to distance from baseline towards an XLO target. There is no sensible way to equate or compare the magnitudes of the excursions observed across these two conditions. I will not consider this experiment in detail. Hypothesis 1 and 2 are built out of the misleading comparison of two incomparable conditions. The result of Expt 2 is summarized in the abstract as "Experiment 2 found that downward convergent changes in F0 could not be increased by the use of an accompanist voice with an even lower F0." That cannot be discerned by comparing XLO with HI from Expt 1. It could be examined by comparing, preferable within subject, LO vs XLO. The discussion on p. 28 ("However, the fact that this convergence was greater for high pitched versus low pitched accompanist voices raises questions surrounding the correct interpretation of these F0 changes." is not well constructed, given these problems. The introduction of a vague suggestion that the "process of synchronization itself" might underlie these changes looks like a red herring. Has anyone suggested this? At this point, the authors (correctly) note that shifting down may not be entirely comparable to shifting up. That is absolutely to be expected, given the complexities of the underlying physiology, and has nothing to do with synchronization.

The lead into Experiment 3 is motivated thus: "In order to test whether any portion of the upward movement in F0 seen during choral speech with a high pitched voice in Experiment 1 can be explained by effortful speech during the act of synchronisation, Experiment 3 measured F0 changes during a visual choral speech condition." The condition that is being motivated is not hard to motivate. We have as yet little empirical study of joint speech. The role of vision has only been touched upon once, and crossmodal stuff will be of interest to speech scientists under these conditions. There is no need to bring in the spectre of "effort" which is undefined, and undefinable. The prior art in speech shadowing is very remotely related to the present conditions, and does not provide strong motivation.

The large number of exclusions necessary for obtaining a clean sample with 10 participants per condition (13 + 5 + 2 + 1 excluded) needs to be mentioned here. The present conditions for research are highly constrained. Under such conditions, it is more necessary than usual to avoid over-interpreting data. A study like this is valuable, but not if it overreaches. When so many have to be

excluded, it seems that it would be wise to state clearly that this is pilot work that can guide future experiments, and no further.

Here, the first hypothesis is fine. It emerges naturally from the design of the experiment.

The statistical analysis is overly complex. When you are looking for simple effects, they should be treated as simple effects. T-tests or their multi-condition non-parametric variants would suffice.

Hypothesis 2 is concerned with magnitudes. See above.

General discussion. At this point, I think it better not to discuss the argument in detail. My points above mean that the experiment needs to be presented differently. There are simple and interesting effects found.

The complexity and over-elaborate analysis hides the basic findings. The basic findings are to be found in the three tables that report convergence, nothing, divergence:

```
Expt 1

HI        6.    2.    2.
LO        4.    3.    3.

Expt 2

XLO.      4.    3.    3.

Expt 3

VIS.      1.    7.    2.
AUD.      8.    2.    0.
A/V.      8.    0.    2.
```

A few t-tests or non-parametric equivalents would suffice to underscore these findings. They do not speak clearly. There was less convergence than expected.

The discussion of the significance of these results for specific models is superfluous. It is worth noting in passing that speech science done using neurocentric, individualistic, representational models has ignored or failed to even see joint speech. A dynamical perspective in which coupling and degrees of non-independence can be represented makes it immediately of interest.

So I recommend making this a much smaller paper, with greatly reduced statistical analysis, with clear presentation of what are interesting (if not univocal) results, and keeping an eye towards the design of future experiments under more felicitous conditions.

Fred Cummins

References

Wang, B. and Cummins, F. (2003). Intonation Contours in Synchronous Speech. Abstract of poster presented at ASA, Austin, 2003. Abstract is here: https://asa-scitation-org.ucd.idm.oclc.org/doi/10.1121/1.4778142

Cummins, F. (2004). Synchronization among speakers reduces macroscopic temporal variability. 26th Annual Meeting of the Cognitive Science Society, pp. 304-309.
