# Supplementary material for: Convergence in voice fundamental frequency during synchronous speech
Source: PLoS One. 2021 Oct 21;16(10):e0258747. doi: 10.1371/journal.pone.0258747 (PMC8530294; doi:10.1371/journal.pone.0258747)
Supplement: Attachment — Submitted filename: Response to Reviewers.docx [file pone.0258747.s005.docx]

**Response to Reviewers**

We would like to thank the editor and all three reviewers for your constructive comments on this manuscript. We have incorporated these suggestions into the revised manuscript, and feel that they have improved the quality of the paper. Please find below our responses to each of the points raised. When reading the revised manuscript and responses to your comments, please note that experiments 1 and 2 from the original paper have now been combined into a single analysis, and so references to ‘Experiment 1’ include the 3 conditions high, low and extra-low, whereas references to ‘Experiment 2’ now refer to the comparison of the audio-only, visual-only and audiovisual conditions (previously Experiment 3). Page and line numbers referenced reflect those in the revised manuscript, with tracked changes shown.

During the process of revising the manuscript, it came to our attention that there was a coding error in the calculation of the confidence intervals for the zero-intercept model used in Experiment 2 to test whether F0 changes in the visual-only condition were significantly different from zero. With this error now fixed, the confidence intervals for the visual-only condition now do contain zero, meaning that at the group level, F0 changes in this condition were not significantly different from zero. We have therefore updated our reporting and interpretation of this result accordingly:

- Experiment 2 Results (page 47, lines 1096-1110): “*Confidence intervals for the audio-only and audiovisual conditions did not contain zero (all CIs [>.15; <1.9]), indicating significant upward changes in F0 from solo reading baseline to synchronous speech. Conversely, confidence intervals for the visual-only condition did contain zero (-0.87; 0.18), indicating that F0 change from solo reading to synchronous speech was not significantly different from zero. This pattern was also seen in the significance of the fixed effects in the zero-intercept model; the effect for the visual-only condition was not significant (β = -0.348, t(30.36) = -1.35, p = .188), whereas effects for the audio (β = 0.675, t(30.38) = 2.61, p = .014) and audiovisual conditions (β = 1.285, t(30.37) = 4.96, p < .001) were both significant.*”
- Experiment 2 Discussion (page 50, lines 1175-1184): “*It should be noted that there was some evidence of changes in F0 in the visual-only synchronous speech condition; in contrast to the convergent upward changes in F0 seen in the audio-only and audiovisual conditions to the high F0 voice, a number of participants in the visual-only condition showed a tendency to slightly decrease their voice F0 (demonstrated in the high number of participants showing apparent ‘divergence’ in Table 2). This could be driven by a slowing of speech rate and production of flatter intonation patterns during synchronisation (which would affect our median measure of F0 across each sentence). These changes in F0 were however not significant at the group level in our zero-intercept model.*”

**Comments from the editor**

1. Reviewer 1 suggests streamlining the analyses and I concur with this suggestion. In fact, it seems to me that there is some redundancy that leads to conflicting results, especially in Experiment 3: While the difference between audio-only and audio-visual was not significant for difference scores in the first analysis it was so when both phases were considered separately in the second analysis. This may be due to spurious effects to do with the F0 values in the SR phase. In my view, there should only be one set of analyses for each experiment performed on adjusted (more on this below) differences between SR and CS with full random effect structure (i.e. not just random intercepts of participants and sentences but also random slope of Condition by sentences).

- In view of reviewer comments, we have decided to remove the F0 distance measure and analyses, in order to avoid confusion associated with this measure and to streamline our analyses. We now report a single set of analyses for each experiment performed on a measure of F0 change from solo reading to synchronised speech (also now quantified in semitones, as requested). In Experiment 2 (previously named Experiment 3), we have now modelled the LMM with a full random effects structure as suggested by the editor. However, for Experiment 1 the random slope of Condition x Sentence was excluded, due to a failure of the model to converge with this effect.

2. The theoretical limitations associated with the use of pre-recorded voices with respect to how informative the study can be for joint action models should be clearly acknowledged.

- We have now expanded on the section in the general discussion that highlights the limitations of the use of pre-recorded voices, to acknowledge this specific limitation in terms of the relevance of the study findings to models of joint action (see pages 59-60, lines 1406-1414):
  - *“Importantly, the unique aspect of live synchronisation is that both talkers can simultaneously align their speech productions with one another, rather than convergence being a one-way-process. This absence of two-way interaction in our design thus limits the informativeness of this study for models of joint action, instead constraining our interpretation to individualistic representational models. It will be important for future research to follow up these preliminary findings with an in-person study of synchronous speech using live interacting pairs, in order to contribute to our understanding of joint speech production.”*

3. Aim for terminological coherence with the literature in the use of ‘choral speech’ and acknowledge other terms and their definitions.

- We have now changed our use of the term ‘choral speech’ to ‘synchronous speech’ as suggested by Reviewer 1 in order to achieve consistency with other literature. We do however acknowledge that in the stuttering literature this is often referred to as choral speech (see page 4, line 81-83):
  - *“Strikingly, synchronous speech (or “choral speech”, as it is often referred to in this literature) can temporarily reduce the occurrence of such dysfluent events…”.*

4. If you streamline the F0 analysis as suggested you may want to consider analysing F0-range. Indeed, far-reaching theoretical conclusions about convergence in general based on just one parameter may not be warranted.

- We acknowledge that it will be important for future work investigating convergence during synchronous speech to examine a wider range of acoustic and phonetic measures than we have investigated here, under more favourable conditions where in-person audio recording is possible. We have added a sentence to our discussion to reflect this (see page 58-59, lines 1385-1389):
  - *“Although several steps were taken to try to mitigate potential effects of this variability, this study would benefit from replication in a laboratory environment where specialist equipment could be used to record participants’ voices in person. This would further allow for a wider range of acoustic and phonetic measures to be examined, such as formant frequencies.”*
- For the current paper, due to reviewer 1’s concerns that the main findings were previously not standing out or speaking clearly in the originally submitted manuscript, we are reluctant to add in more measures that may split the focus of the results and make interpretation of the main effects more complicated. Our focus on median F0 only, being the parameter that was specifically manipulated in our accompanist recordings, parallels the approach taken in a recent similar paper by Aubanel and Nguyen (2020, *PLOS ONE*) - they also manipulated the F0 of an interlocutor’s voice as heard by the participant and therefore only looked at changes in median F0 across turns that mirrored these changes.

5. I agree with Reviewer 1 that the Hypotheses do not flow directly from the Introduction and need to be better motivated early on. This may then remove the need to restate them for each experiment.

- We have now added new content to the end of the introduction, to try to make the motivations behind each of the two experiments clearer from the start (see pages 10-11, lines 221-244):
  - *“In order for us to advance our understanding of these effects, the current study aimed to test whether 1) speakers given simultaneous other-voice feedback during speech – by speaking in synchrony with another talker – show changes in their vocal acoustics, and 2) whether these changes are specific to the external acoustic feedback, rather than being driven by some other aspect of speech synchronisation. In order to do this, experiments are needed which test for acoustic convergence during synchronous speech that is specifically dependent on the auditory properties of the accompanist talker. In Experiment 1, we test this by measuring changes in F0 during synchronous speech, specifically testing for upward changes in F0 during synchronisation with a higher-pitched accompanist, but downward changes in F0 in participants who synchronised with a lower-pitched accompanist. Furthermore, in order to test theoretical frameworks that prioritise auditory feedback for speech motor control, it is important to confirm that these convergent changes are indeed primarily driven by acoustic properties of the other talker’s voice, and are not equally generated by other types of input like visual speech information. We test this in Experiment 2, in the inclusion of a visual-only synchronous speech condition (where participants could see but not hear the accompanist), as well as an audiovisual condition (where participants could both see and hear the accompanist). Across both experiments, we predicted that participants would show changes in F0 that were dependent on the F0 of the accompanist voice they experienced (i.e. they should increase to a high F0 accompanist, decrease to a low F0 accompanist, and remain unchanged during visual synchronous speech). Conversely, if changes in F0 are simply a by-product of synchronisation of speech timing with an external stimulus, we would expect uniform changes in F0 across these different conditions.”*
- We have now also added separate ‘Measures and Hypotheses’ sections for each experiment that come just prior to reporting of results. These sections outline and explain our hypotheses for that experiment (for Experiment 1, see pages 21-22 lines 495-511; for Experiment 2, see page 43, lines 1010-1026). These now focus only on our predictions for how F0 change from solo reading to synchronous speech will differ across the conditions in each experiment.
  - E.g. for Experiment 1 on page 21: *“Our central hypothesis across all experiments was that F0 changes during synchronous speech would be specific to the accompanist voice experienced. For this experiment, we therefore predicted that participants in the high F0 accompanist condition should show significant increases in F0 from solo reading to synchronous speech, while participants in the low and extra-low F0 accompanist conditions should show significant decreases in F0. At the group level, we predicted that this expected difference in the direction of F0 changes across the conditions would result in a significant group difference, in which the predicted negative F0 change in the low and extra-low conditions would be significantly lower (i.e. more negative) than the predicted positive F0 change in the high condition.”*

6. The alternative – that F0 convergence is a by-product of trying to converge in speech rate, presumably due to greater effort, should be theoretically better motivated.

- We have now provided more background to the idea that increased speaking effort during synchronised speech may lead to global changes in F0 that are not related to vocal convergence; this is in the discussion section following experiment 1 that motivates the inclusion of a visual-only choral speech condition in experiment 2 (previously experiment 3) in order to directly test this idea (see page 30, lines 686-709).
  - *“The rationale for including conditions designed to induce both upward and downward convergent shifts in F0 was to determine whether measured changes in F0 during synchronous speech might simply be driven by the act of speech synchronisation itself. That is, it is possible that the act of adjusting the timings of one’s speech so as to be in synchrony with an external stimulus could itself induce systematic global changes in F0. There is evidence that speaking conditions associated with increased effort can lead to global changes in F0. For example, when speaking in adverse listening conditions (e.g. background noise or babble), speakers adopt a ‘clear speech’ speaking style that is accompanied by global changes in F0; Hazan and colleagues (52) reported global increases in mean F0 that did not serve to enhance specific phonological contrasts, suggesting they could reflect a by-product of increased speaking effort. In the field of vocal convergence, a study by Kappes, Baumgaertner, Peschke and Ziegler (53) found reduced convergence in F0 in a speech shadowing task (where participants had to repeat non-words with as minimal a delay as possible, even during the ongoing stimulus presentation) compared to a delayed repetition task; they attributed this to an overall tendency to increase F0 due to increased speaking effort in the shadowing task. In a similar way, synchronous speech could have a global effect on F0 due to the increased effort associated with timing the pace of one’s speech with an external stimulus. Our demonstration in Experiment 1 that F0 changes during synchronous speech appeared specific to the acoustic characteristics of the accompanist voice experienced goes some way to addressing this concern. However, a more direct test of this idea can be achieved through the use of a visual synchronous speech condition…”*

7. Clarify baseline comparison between groups prior to experimental manipulation so that the condition label terminology does not become confusing. Also, please aim for consistency of labelling the solo reading phase either as that or as baseline.

- We understand that the original phrasing of this comparison was misleading; we have now rephrased this to reflect the fact that this is a check of equivalence between groups before they were exposed to our experimental contrast e.g. page 24, lines 550-554:
  - *“To check the comparability of our three groups, we first checked that the average F0 of participants during the solo reading baseline task did not significantly differ between the three groups (i.e. before they were exposed to our experimental manipulation in the synchronised speech task). A one-way ANOVA found that average F0 at solo reading baseline did not differ between groups (F(2, 27) = 0.087, p = .917).”*
- To ensure that there is no confusion as to the term ‘baseline’, we now clearly define the solo reading task as our baseline task in the General Methods section one page 13, lines 288-292: *“The first experimental task for all experiments was a solo reading task… This was treated as a ‘baseline’, in order to measure the participant’s F0 when not exposed to another voice/engaging in synchronous speech.”* We have now also ensured consistency in the use of the term “solo reading baseline” throughout the manuscript, which we hope will be a less confusing term than “baseline” on its own.

8. Clarify what distances you are reporting on. In addition to Reviewer 1’s comments on defining the differences I would caution that absolute differences in Hz obscure the non-linear nature of pitch perception so in my view differences should be reported using a log-transformed measure such as semitones.

- Due to reviewer 1’s concerns about our F0 distance measure and the editor’s suggestion that we streamline our analyses, this measure has now been removed from the manuscript. We have now also updated our measure of F0 change to be expressed in semitones rather than raw Hz, as requested.

9. Introduce clear labels for all factors early on. It took the Reviewers and myself a while to figure out what ‘phase’ refers to – this should have been clearly stated when the design is explained.

- Our apologies that this was not clear in the original manuscript. We have now removed the analysis of F0 distance across the two tasks/phases of the experiment, and so this is no longer relevant.

10. Consider a direct comparison between XLO (Exp 2), LO (Exp 1) and HI (Exp 1) conditions, essentially merging Experiments 1 and 2 into a joint analysis, while maintaining transparency in reporting the order in which they were carried out.

- As requested, we have now combined experiments 1 and 2 into a joint analysis comparing these three conditions, while highlighting in the methods section that the extra-low condition was tested after the data for the high and low conditions had been collected and analysed. e.g. see pages 16-17, lines 379-385:
  - *“Initially, two conditions were tested corresponding to these high and low F0 conditions. After this data was collected and analysed, a further ‘extra-low F0’ condition was tested, in which participants synchronised with an accompanist voice with an even lower F0. This was added after observing the distribution of solo reading baseline F0 in the initial sample tested with our* a priori *designed conditions (see Results). For simplicity, we report here on the results of a single data analysis incorporating all three conditions.”*
- See also page 24 lines 561-568 in the results section:
  - *“From these descriptives, it can be seen that the distribution of F0 values measured during solo reading baseline in the low F0 group overlaps with the average F0 of the accompanist voice to which they were subsequently exposed in the synchronous speech task (170Hz). Since it was our aim to use accompanist voices whose average F0 was far away from that of our sample at solo reading, this motivated the design of our additional extra-low F0 accompanist condition. In this condition, the accompanist voice’s average median F0 (140Hz) was substantially lower than the distribution observed in our initial sample.”*

11. In the figure captions, please state clearly what the abbreviations mean and what the units of the differences are (semitones I would hope in a revised version).

- The figure captions now clearly explain what is being reported in the graphs *e.g. “Fig 2: F0 change across trials in high, low and extra-low conditions. Graph shows average change in F0 (in semitones) from solo reading baseline to synchronous speech for each trial of the task, averaged across participants in high, low and extra-low conditions. Shaded areas around lines indicate standard error.”* The figures now contain no abbreviations since the F0 distance measure has been removed.

12. Finally, a small issue I noticed – in line 103, shouldn’t it say ‘if listeners tend to chose B more often…’ ?

- The explanation of the AXB procedure in the original manuscript was not quite correct; this has now been amended to concur with the explanation of this procedure given in Pardo (2013; *Frontiers*) (page 5, lines 102-109):
  - *“…or through the use of perceptual judgements by independent listeners in AXB designs (8). In the latter, an external set of listeners are asked to judge whether a participant’s utterance during an interaction (stimulus A) sounds more similar to the model utterance from their interlocutor (stimulus X), compared with that participant’s baseline utterance when speaking alone (stimulus B) – if listeners tend to choose A more often than B as the stimulus most similar to X, this suggests that the participant’s speech has moved perceptibly toward the interlocutor when speaking with them.”*

**Reviewer 1:**

1. The manuscript reports a series of experiments examining F0 changes during synchronous speaking. There has been little work reported on changes to F0 under these conditions, so there is a gap here to be filled. The work is all carried out under the onerous conditions of the pandemic, which has constrained what can be done greatly, and it is to be hoped that this is regarded as preliminary to a less constrained study. Overall, the work reported here should be a valuable contribution to a growing literature that can guide further inquiry. As the manuscript stands, there are issues with how results are reported that require substantial revision. I am of the view that this paper should be much much shorter, with less statistical analysis. Analysis should make clear the very simple effects found, and should avoid complicating matters with wrongly used technical corrections and discriminations.

- We have carefully considered all the Reviewer’s comments and made substantial changes to the manuscript, including removing our previously included ‘F0 distance’ measure and its associated statistical analyses, as well as combining Experiments 1 and 2 into a single Experiment 1 with one set of statistical analyses on the F0 change measure (see responses to specific comments below for more details). We believe this now makes the manuscript a much clearer and more compact paper, with results that are easier to interpret.

2. One obvious limitation of the constraints is the uncontrolled nature of the recordings which are done by subjects using their own computers and microphones. This is addressed sensibly as far as possible, and the inclusion of the Higgins Pitch test and the vigilance check go some way to ameliorating the limitation. It seems to this reviewer that usable F0 data might be obtained in this fashion.

A second limitation is the use of pre-recorded voices with which subjects are asked to align. The difference between coordination with a recording, and reciprocal coupling with a live voice is an extremely important one (as we found in Jasmin et al, 2015, and elsewhere). It is important for many reasons, which the authors are aware of. The motivating interest in choral speech support for stutterers makes this somewhat less of an issue, but it greatly reduces the likelihood that our understanding of joint speech production will be furthered by this study. The use of recordings, rather than live co-speakers, has the additional effect of supporting interpretation in terms of individualistic representational models, as here, rather than dynamical embodied models, as becomes more attractive when the intersubjective situation of co-speaking is employed. This thrust towards one kind of model rather than another is not acknowledged.

- We agree that the use of pre-recorded voices, although necessary due to the unique conditions in which this study was run, is a major limitation of the present study. We indeed already highlight in the Discussion the ways in which synchronisations with live and pre-recorded voices represent two very different contexts. We have now expanded on this to underscore the limitations this therefore places on interpretation of our findings in terms of models of joint action, and the need for future research to follow up these preliminary findings with in-person studies of live interacting pairs (pages 59-60, lines 1406-1414):
  - *“Importantly, the unique aspect of live synchronisation is that both talkers can simultaneously align their speech productions with one another, rather than convergence being a one-way-process. This absence of two-way interaction in our design thus limits the informativeness of this study for models of joint action, instead constraining our interpretation to individualistic representational models. It will be important for future research to follow up these preliminary findings with an in-person study of synchronous speech using live interacting pairs, in order to contribute to our understanding of joint speech production.”*

3. Which leads naturally to the term "choral speech". Having specialized in all forms of collective speaking over many years, I have tried to encourage others to adopt a coherent terminology. The original work on stuttering did indeed use the term "choral speech". This term otherwise normally refers to the performative group recitation of poems and the like performed, often competitively, by children in South East Asia and in Ireland (at least). The term Synchronous Speech, I have argued ought to be reserved for the kind of laboratory experiment done here, while the umbrella term Joint Speech has the useful function of covering all occasions in which multiple people utter the same sounds at the same time. I cannot enforce terminological uniformity, but I can plead with my colleagues that these considerations should encourage us to use terms in a manner that is well aligned. The original work on stuttering long predates these additional concerns, and it is to be hoped that the field will become more consistent over time.

- We completely understand the argument for promoting consistency in the terminology used for collective speech behaviours, and have now changed our use of the term ‘choral speech’ to ‘synchronous speech’ as suggested. We do however acknowledge that in the stuttering literature this is often referred to as choral speech (page 4, lines 81-83):
  - *“Strikingly, synchronous speech (or choral speech as it is often referred to in this literature) can temporarily reduce the occurrence of such dysfluent events…”*

4. There is little prior work on F0 and synchronous speech. The authors note Cummins (2000), which noted that pitch range is reduced in synchronous speaking conditions. They have not noted Cummins (2004) which documented lower intonational variability (peaks and valleys) under synchronous speaking conditions. Understandably absent is Wang & Cummins (2003) which is just a published abstract, but it examined details of intonation contours in a tonal language, which brings me to a substantial point: the only variable employed here is the median F0 over a sentence length utterance. This variable is suited to a coarse examinination of phonetic convergence, as here, but it omits a lot of detail that is possibly also of interest, including range, variability, and slope. I would venture to suggest that a more fulsome examination of F0 under these conditions ought to employ more detailed measurements, if they are to be of use in adjudicating the claims of competing models.

- We have now added references to Cummins (2004) and Wang & Cummins (2003) on page 9, line 194.
- We agree that it will be important for future work investigating convergence during synchronous speech to examine a wider range of acoustic and phonetic measures than we have investigated here, under more favourable conditions where in-person audio recording is possible. We have added a sentence to our discussion to reflect this (pages 58-59, lines 1385-1389):
  - *“Although several steps were taken to try to mitigate potential effects of this variability, this study would benefit from replication in a laboratory environment in which specialist equipment could be used to record participants’ voices in person. This would further allow for a wider range of acoustic and phonetic measures to be examined, such as formant frequencies.”*
- For the current paper, due to the Reviewer’s concerns that the simple effects were not clearly presented in the original manuscript due to too many statistical analyses, we are reluctant to add in more measures and analyses that may steal focus away from the main results. We chose to focus on median F0 only for measuring convergence, as this was specifically manipulated in our accompanist recordings. This parallels the approach taken in a recent similar paper by Aubanel and Nguyen (2020, *PLOS ONE*), where they also manipulated the F0 of a partner’s voice as heard by the participant and therefore only looked at convergent changes in median F0 across turns.

5. Experiment 1: Here, convergence towards the recorded target is observed both downwards and upwards. That result was to be expected and is nice to see. I had great difficulty with the manner in which the results are reported, and I suggest that a substantial rewrite is needed if the important features observed are to be brought forth, and confusing details relegated to the background.

A pragmatic choice to use only female speakers was made. This seems fine and justifiable. However it does not remove the messiness of human physiology from the equation and propel us into a metric space in which meaningful distances can be measured. Each speaker has her own larynx, her own idiosyncrasies of movement, and can be assumed, in a first approximation, to have something like a center, or mid-point, from which excursions upwards and downwards will require speaker- specific effort. It cannot be assumed that each speaker is free to raise or lower their F0 to an arbitrary extent, or that different speakers have similarly "elastic" pitches. In the ensuing analysis, the authors assume that the median F0 obtained in the solo speaking task is, for each speaker, a kind of "baseline." I'm afraid that term gave me the greatest difficulty, and I will try to explain why. (It took very many readings to discover what is being reported here... I may still be misinterpreting things.)

- We apologise that use of the term ‘baseline’ was unclear; we now clearly define the solo reading task as our baseline task in the General Methods section on page 13, lines 288-292: *“The first experimental task for all experiments was a solo reading task… This was treated as a ‘baseline’, in order to measure the participant’s F0 when not exposed to another voice/engaging in synchronous speech.”* We have also now ensured that the term ‘baseline’ is never used on its own, but always clarified as ‘solo reading baseline’.
- We believe that the treatment of the solo reading task as a baseline task is justified, and accords with other literature on vocal convergence. For example, a recent study by Lee, Goldstein, Parrell & Byrd (2021, *PLOS ONE*) compared participants’ productions between a solo speech task and an interactive task, and refer to variables measured from the solo speech utterances as “baseline” measures e.g. “Baseline CoV: the individual variability inherent to an individual speaker’s solo speech. Baseline CoV was assessed by comparing moving CoV values of the two dyad members in their solo maze conditions (before the cooperative maze task).”

6. These speakers are now given the task of speaking along with recordings that have been prepared to have relatively high or low median F0. The experimental result obtained is evidence that when speaking in synchrony there is a shift in median F0 upwards or downwards, depending on condition. That is the result, but it is hidden in a barrage of non-results that I will now look at.

The t-tests (p. 18, lines 412ff) document this shift. They are done on a per-subject basis, which is entirely appropriate.

The difficulties start on p. 19, line 427. "At the group level, average baseline F0 (at solo reading) did not differ between high (M = 193.86, SD = 25.28) and low (M = 198.21, SD = 26.07) F0 conditions (t(17.98) = -0.379, p = .709)." This took me forever to decode. If I understand correctly, this is a comparison of the solo speech from two groups, given the between subjects design. Its purpose is to verify that the between subjects design is reasonably justifiable. Large differences here might lead one to question the equivalence between groups, threatening the experimental design.

However the reporting is done in terms of the "high" and "low" conditions. These are utterly irrelevant and misleading. It is true that each group will go on to speak with recordings that are high or low, but the analysis here is of the solo recordings only. The experimentally manipulated recordings play no role. This should be reported as a check of equivalence between groups, prior to exposure to experimental contrasts. It should not be framed in terms of those experimental contrasts. The manner in which this is reported destabilized the notion of a baseline for me and led to hours of alternative consideration as to what baseline might be meant. I strongly suggest not using that term, but if it is used, it should not be subjected to this kind of problematic confusion with irrelevant factors (High and Low are irrelevant here. It is simple inter-group comparability that is being tested.)

- We understand that the original phrasing of this comparison was misleading; we have now rephrased this to reflect the fact that this is a check of equivalence between groups before they were exposed to our experimental contrast (page 24, lines 550-554):
  - *“To check the comparability of our three groups, we first checked that the average F0 of participants during the solo reading baseline task did not significantly differ between the three groups (i.e. before they were exposed to our experimental manipulation in the synchronised speech task). A one-way ANOVA found that average F0 at solo reading baseline did not differ between groups (F(2, 27) = 0.087, p = .917).”* Note that this is now a one-way ANOVA that compares the low, high and extra-low F0 groups, since these have now been combined into a single analysis, as requested by the editor.

7. A second problematic report follows, on Line 433: "However, distance in F0 from the accompanist voice at baseline did differ between conditions." This still makes my head spin and makes the "baseline" more mysterious yet. The syntax alone does not make it clear who owns the baseline, and it suggests that "the accompanist voice at baseline" is a thing. It is not a thing, and I lost more time over this. My best interpretation of this assertion is that the degree of convergence observed in each group was not identical, and this rather simple result is augmented by the much more problematic claim that there was "a greater F0 distance at baseline in the high condition (M = 70.56, SD = 25.13) compared to the low condition." This is not only confusing, but confused. The distances that are summoned to warrant the terms "greater" and its assumed "lesser" for the low condition, are conjured up by the experimenter. They are not observations, but design decisions. The same experiment could have been run with target recordings that had been manipulated to have higher or lower values. If we leave the high target the same, but drop the low target by 50 Hz, the result will be different, not because of anything the subjects did, but because we designed things that way. Again, I may be misunderstanding things, but if I am, something is still clearly amiss that such misunderstanding after diligent effort should be possible.

My interpretation is supported by the ensuing claim (line 436...): "This indicates that participants in the high condition started the task with an F0 that was further away from their corresponding accompanist’s F0 compared to those in the low condition with their respective accompanist’s F0." Is this not obviously just the arbitrary values the authors chose to place the F0 of the accompanist at? The phrase "started the task" confused me further. There is no analysis here of a temporal progression or time series, which is what those words suggest. Here the authors seem to be acknowledging that "well, we just put this here" and reporting that as a result. I understand that the "baseline" is different for each participant, and so they might differ in where their zero point lies between the two extremes, but the extremes are set by the experimenter.

- The explanation of the F0 distance measure was included in the general methods section of the original manuscript under the heading “Acoustic Analysis”, however we think this has been (understandably) missed by reviewers since it was not repeated for the individual methods sections of each experiment. The reviewer is correct that this measure reflects the difference between the median F0 values of the participant across each task and the median F0 values of the manipulated recordings. That is, the F0 values of the participant at solo reading and synchronous speech are each separately compared to the F0 values of the single set of recordings for the accompanist (the reviewer is quite right that there is no equivalent ‘baseline’ or ‘solo reading’ recordings for the accompanist); comparison of the two sets of distances can then be used to give a so-called ‘difference-in-distance’ measure. Our rationale for including this measure was that we wanted a measure that not only reflected how F0 values changed from solo reading to synchronous speech, but further reflected to what extent these F0 changes actually brought the participant closer to the F0 of the accompanist voice. That is, presumably if a participant actually converged to the accompanist, the difference between their synchronous speech F0 values and the F0 of the accompanist should be smaller than the equivalent distance measured during solo speech (when the participant was not exposed to the accompanist voice). This measure was thus meant to quantify how far away from the accompanist F0 the participant was ‘at baseline’ (at solo reading) and then how close they were able to move during convergence (during synchronous speech). We understand that of course this measure is therefore partially dependent on the design decisions of us as the experimenters in the sense that we set the average median F0 of the accompanist. However, we are of the opinion that this is not completely arbitrary as this was the F0 of the accompanist the participant actually experienced, and there is evidence to suggest that the initial acoustic distance between two interlocutors does have an effect on the extent to which they converge (see references on page 29, lines 666-670 in the manuscript). We do however also acknowledge here that in some cases, this apparent relationship may be an artefact of the difference-in-distance measure itself, as demonstrated recently by Priva and Sanker (2019, *Laboratory Phonology*) and MacLeod (2021, *Journal of Phonetics*).
- Overall, in response to comments from this reviewer and the editor, and given these recent demonstrations of problems with distance measures in the above papers, we have decided to remove this F0 distance measure from the paper, and instead focus on the F0 change measure for our analyses. We hope that this will make the findings easier for the reader to interpret. We have however added some new plots for each experiment showing the difference in semitones between each participant’s F0 and the accompanist F0, separately for the solo reading baseline and the synchronous speech tasks (see Fig 1 and 4). These provide a visual illustration of convergence, and enable the reader to clearly see for each participant how far they changed in F0 from solo reading to synchronous speech, and further, whether this change actually brought them closer to the accompanist F0. We note, however, that all inferential statistical analyses are carried out on the F0 change measure (in semitones) only.

8. Then the first hypothesis is stated: "Hypothesis 1: Change in produced F0 values during choral speech will be significantly different between high and low F0 conditions". When a hypothesis is stated, it must first be motivated and argued for. This has not happened, and this appears out of the blue. Furthermore, upon examination, it has the same problem just mentioned. That there might be convergence towards the HI and LO targets is clear, agreed, and found. But to express this as a magnitude that can be quantitatively compared across conditions raises two substantial problems. 1: as noted, the targets have been somewhat arbitrarily set by the experimenters, so any asymmetry in degree of convergence is at least partly built in, not found. 2: The difference alluded to in the hypothesis is metric. The voice is not an abstract thing. Each speaker will have personal limits on how far, and fast, they can modulate their voice. If we were to (wrongly) suggest that subjects are actually striving to reach the value of the accompanist, it would surely be clear that this represents a different challenge for each speaker, depending on their capacity for vocal modulation. Of course, many experimental designs specifically set out to wash out such inter-participant differences by averaging, but that is to treat the voice as if it were itself abstract. The authors chose not to include men, for their inclusion would make it obvious that averaging is inappropriate. The problem does not go away with reduction to a single sex.

While the latter problem may be unavoidable, it matters, and the hypothesis that is conjured up out of the blue needs to first consider such factors, and to put forward arguments relating not only to direction, but to magnitude, which is not straightforwardly measurable. The LMM analysis that follows is intended to make manifest metric differences, and thus to interpret magnitudes. But there are no objective magnitudes possible when the experimenters design not only the axis of measurement, but its underlying topology. Consider an analogy. I want to see how high a bunch of students will jump, so I hold out a bar and encourage them to jump over it. The bar is higher than they can jump, but they valiantly try. Another teacher does the same experiment but makes the bar much higher. Students faced with the higher target try harder, and their jumps have a different distribution than my students. The analogy is not great, but perhaps it can illustrate how any magnitudes measured under such circumstances depend in a serious way on the choices of the experimenter beforehand.

Despite all these issues, the finding seems perfectly clear and could be demonstrated in a simpler manner and with less possibility for confusion. "Therefore, as predicted, F0 change from baseline was positive in the high condition, but negative in the low condition. This suggests that the acoustic properties of the accompanist’s voice (specifically, F0) did affect the nature of the change in the participants’ voices at choral speech." This is clear, correct, and of interest. It does not invoke magnitudes.

- We have now added separate ‘Measures and Hypotheses’ sections for each experiment that come just prior to reporting of results, that outline and explain our hypotheses for that experiment (for Experiment 1, see pages 21-22 lines 495-511; for Experiment 2, see page 43, lines 1010-1026). These now focus only on our predictions for how F0 change from solo reading baseline to synchronous speech will differ across the conditions in each experiment.
- For Experiment 1, we have tried to clarify that our prediction here for the comparison between F0 change values in the high versus the low F0 conditions is in fact not concerned with the magnitude of convergence observed. This would be the case if we were to compare *absolute* F0 change between the conditions. Conversely, here our measure of F0 change preserves the sign of these differences; we are therefore expecting to see positive F0 change values in the high F0 condition, and negative F0 change values in the low and extra-low conditions, which should result in a significant group difference (high versus low). This hypothesis and statistical test is therefore concerned with the *direction* of F0 change observed in the two conditions, and not with the absolute magnitude of convergence (see pages 21-22, lines 502-511):
  - *“Our central hypothesis across all experiments was that F0 changes during synchronous speech would be specific to the accompanist voice experienced. For this experiment, we therefore predicted that participants in the high F0 accompanist condition should show significant increases in F0 from solo reading to synchronous speech, while participants in the low and extra-low F0 accompanist conditions should show significant decreases in F0. At the group level, we predicted that this expected difference in the direction of F0 changes across the conditions would result in a significant group difference, in which the predicted negative F0 change in the low and extra-low conditions would be significantly lower (i.e. more negative) than the predicted positive F0 change in the high condition.”*

9. Hypothesis 2 is then provided: "Hypothesis 2: F0 distance will be lower at choral speech compared to solo reading in both high and low conditions". Once more, this hypothesis is created out of air. There has been no argument to motivate or justify it. I find it uninterpretable. What distance is meant? The figures do not have any captions, despite the problematic practice of the journal of placing them at the end of the manuscript. When that is done (sigh) there should at least be a list of figure captions. The abbreviations SR and CS I have reverse engineered to deduce they mean Solo Reading and Choral Speech. Given that reading is not at stake here, the SR abbreviation (if I am guessing correctly) is misleading. At the bottom left of Fig 3 the two words "phase" and "condition" occur. Do they belong together? "Phase" is a word with a lot of meanings. What could it possible mean here? And most importantly of all, the Y-axis is F0 distance, but nowhere does it say distance between what and what.

The hypothesis that came from nowhere is expanded on thus (p. 20, line 471...): "To test whether the distance between participant and accompanist F0 values was smaller at choral speech compared to solo reading, an LMM analysis was used on F0 distance values." This is unclear. The accompanist does not provide solo readings (again, "reading" is misleading, when speaking is at issue). If I try to rephrase this, I think it means that median F0 values measured in the choral speech condition are displaced towards the F0 values of the manipulated recordings. I did not use the word "distance" in that reformulation. Perhaps I have misunderstood, but that seems to me to be the sense.

I have no desire or skill to get into the weeds of debating the specific statistical tests used, but they should be testing something meaningful, and something that we have been primed by argument to expect, and to be able to interpret. An unmotivated or explained hypothesis followed by statistics quickly causes the reader to get lost, and to fail to see what the result obtained is. The LMM model is described thus: "The interaction effect in this model indicated that the difference between F0 distance at baseline and choral speech was greater (more negative) in the high condition compared to the low condition." This statistical test is concerned with the estimation of magnitudes, and as discussed above, the metric that such measurement relies on is not warranted, but arises as a function of the contingent targets set by the experimenters combined with the individual physiology of the participants considered individually. The ensuing dive into Bonferroni correction and whatnot does nothing but obscure the perfectly simple result. An analysis like this achieves nothing, but casts a magical air of scientism over something which is nothing but a simple effect. Interpreting quantitative inferential statistics is difficult at the best of times, but it demands that the scales, magnitudes, and effects be sensible, well motivated, and transparent. That is not the case here.

- This analysis of the F0 distance measure has now been removed from the paper (see previous response to point 7). We hope that the revised manuscript now clearly presents the motivation behind our analyses in our new ‘Measures and Hypotheses’ sections (see response to point 8), such that the reader is well placed to be able to interpret the results of the LMM analyses on the F0 change measure. For Experiment 1, this analysis highlights the simple effect i.e. that F0 change was positive in the high F0 condition, but negative in the low and extra-low conditions.

10. Discussion of Experiment 1: "Overall, experiment 1 found evidence of significant convergence in F0 to an accompanist voice during choral speech, both when convergence required a lowering of F0 (low F0 accompanist condition) and when it required a raising of F0 (high F0 accompanist condition)." This seems fine, secure, and is interpretable. But "However, our analysis found that convergence in F0 was greater in the high F0 condition compared to the low F0 condition." This is uninterpretable and adds nothing. See all my points about measurement, metrics, and physiology.

The authors see some of what I am trying to point to: "However, a flaw in the current experimental design was that the high and low conditions did not start out with an equivalent distance between participant and accompanist F0s at baseline, with a greater distance at baseline in the high F0 compared to the low F0 condition." Yes, but it is not really a flaw. The experiment was designed thus, and there is no way to fix it. Even if you had equal Hz values between baseline and HI and LO, respectively, that numerical equivalence is projected onto a space of flesh, spit and muscle. Equal Hertz is equality within a representational scheme only. There is no fix, any more than one can aspire to collating unbiased data over a non-ergodic, ill-defined, and unknowable population. This certainly has nothing to do with differences between Americans and Brits.

The discussion sets up Experiment 2, more or less in line with my analogy above, by employing a target even more remote from the subject's baseline. That is fine, as far as it goes, but no quantitative conclusions can be drawn from such a modified design. One can ask whether using more remote "targets" (they are not targets, of course) produces a noticeable effect. One can ask nothing sensible about magnitudes, because one does not have command of the underlying space being represented, which is built of flesh, volition, ambition, perceived difficulty, and such intangible quantities.

The comparisons done in Experiment 2 are uninterpretable. The experimenters have no command of the underlying reality, and are forced to compare the HI condition of Expt 1 with the XLO condition of Expt 2. This once more makes the larynx into an abstract thing, to whom up and down are mere directions. Distance from baseline towards a high target are being compared numerically to distance from baseline towards an XLO target. There is no sensible way to equate or compare the magnitudes of the excursions observed across these two conditions. I will not consider this experiment in detail. Hypothesis 1 and 2 are built out of the misleading comparison of two incomparable conditions. The result of Expt 2 is summarized in the abstract as "Experiment 2 found that downward convergent changes in F0 could not be increased by the use of an accompanist voice with an even lower F0." That cannot be discerned by comparing XLO with HI from Expt 1. It could be examined by comparing, preferable within subject, LO vs XLO. The discussion on p. 28 ("However, the fact that this convergence was greater for high pitched versus low pitched accompanist voices raises questions surrounding the correct interpretation of these F0 changes.") is not well constructed, given these problems. The introduction of a vague suggestion that the "process of synchronization itself" might underlie these changes looks like a red herring. Has anyone suggested this? At this point, the authors (correctly) note that shifting down may not be entirely comparable to shifting up. That is absolutely to be expected, given the complexities of the underlying physiology, and has nothing to do with synchronization.

- At the editor’s request we have combined Experiments 1 and 2 into a single joint analysis (while maintaining transparency in the order in which these conditions were conducted). We now do not focus on the differences in magnitude of convergence between high and low conditions. Instead, we simply explain that it was our intention to have high and low F0 conditions with accompanist voices whose F0 was atypically high/low, but that our originally designed low condition involved an accompanist voice with an average F0 that overlapped with the distribution of F0 values in our sample measured at solo reading baseline. This thus motivated the design of our ‘extra-low’ condition, which used an accompanist voice with an average F0 that was far away from the range observed in our sample. This is explained on page 24, lines 561-568:
  - *“From these descriptives, it can be seen that the range of F0 values measured during solo reading in the low F0 group overlaps with the average F0 of the accompanist voice to which they were subsequently exposed in the synchronous speech task (170Hz). Since it was our aim to use accompanist voices whose average F0 was far away from that of our sample at solo reading, this motivated the design of our additional extra-low F0 accompanist condition. In this condition, the accompanist voice’s average median F0 (140Hz) was substantially lower than the range observed in our initial sample.”*
- In the Discussion of Experiment 1, we now further elaborate on our rationale for inclusion of this extra-low condition in relation to recent work demonstrating that differences in ‘starting distance’ between individuals/groups and the model talker can result in artefactual differences in the magnitude of convergence observed; thus it was important to us that all conditions used an accompanist voice which was likely to be similarly far away from the distribution of ‘baseline’ solo reading F0 values in our samples (page 29, lines 662-685):
  - *“As previously explained, it was necessary to design and test post-hoc an ‘extra-low’ F0 accompanist condition in this experiment in addition to the originally planned high and low F0 conditions. This was because the average F0 of our initial low F0 accompanist voice overlapped with the distribution of ‘baseline’ F0 values within our sample as measured during solo reading. Previous research has suggested that the initial acoustic distance between two speakers can affect the degree of vocal convergence observed, with most studies reporting that convergence is facilitated by a greater distance at baseline (47–49), although the reverse pattern has also been reported (10,50). More recently, it has been argued that the former apparent relationship may in fact be an artefact of the way in which convergence is typically calculated, specifically in relation to the use of a ‘difference-in-distance’ measure (comparing the change in absolute distance between a participant and a model talker from before to after exposure) (51,52). In general, Priva and Sanker (51) argued that measurement of convergence can be unreliable when participants’ baseline values are close to the model talker, as the influence of random variability within an individual is likely to overshadow genuine convergent changes, leading to underestimation of convergence or even apparent divergence. This point thus supports our rationale for the inclusion of our extra-low F0 condition, to ensure there was sufficient room for downward convergent changes from solo reading baseline to synchronous speech to be observed.”*
- It is therefore not necessary to discuss differences in magnitude of convergence across the conditions to motivate this extra condition; this was instead added to address a design flaw that meant that our stimuli in the original low condition were not achieving what we intended. However, our main conclusion from the analysis of these three conditions remains the same as before (page 28, lines 653-656): *“Overall, Experiment 1 found evidence of significant convergence in F0 to an accompanist voice during synchronous speech, both when convergence required a raising of F0 (high F0 accompanist condition) and when it required a lowering of F0 (low and extra-low F0 accompanist conditions).”* The paragraph beginning *"However, our analysis found that convergence in F0 was greater in the high F0 condition compared to the low F0 condition"* (page 32, line 745) has now been removed from the Experiment 1 discussion, and we have taken care not to overinterpret or emphasise this apparent difference in magnitude of convergence in upward and downward directions. For example, we have removed the paragraph beginning with the following sentence from the discussion on page 51, line 1188: *“Crucially therefore, the greater convergence to the high versus the low F0 accompanist voices seen in Experiments 1 and 2 cannot be explained by an overall tendency to increase F0 when synchronising speech timing with another speaker.”*

11. The lead into Experiment 3 is motivated thus: "In order to test whether any portion of the upward movement in F0 seen during choral speech with a high pitched voice in Experiment 1 can be explained by effortful speech during the act of synchronisation, Experiment 3 measured F0 changes during a visual choral speech condition." The condition that is being motivated is not hard to motivate. We have as yet little empirical study of joint speech. The role of vision has only been touched upon once, and crossmodal stuff will be of interest to speech scientists under these conditions. There is no need to bring in the spectre of "effort" which is undefined, and undefinable. The prior art in speech shadowing is very remotely related to the present conditions, and does not provide strong motivation.

- We have revised the discussion of Experiment 1, which sets up the motivation for Experiment 2 (previously Experiment 3). This second experiment has two primary motivations: (1) to investigate F0 changes during visual synchronised speech to determine if this induces any systematic shifts in F0 and (2) to investigate whether convergence is enhanced by the provision of visual cues during synchronised speech in an audiovisual condition compared to an audio-only condition. We believe the first motivation is an important empirical question that allows us to directly test whether F0 changes during synchronous speech are specific to the acoustic characteristics of the accompanist voice (our central hypothesis). We have expanded on our rationale for this question by discussing other literature suggesting that global changes in F0 can occur during speaking conditions associated with increased effort (see page 30, lines 686-704):
  - *“The rationale for including conditions designed to induce both upward and downward convergent shifts in F0 was to determine whether measured changes in F0 during synchronous speech might simply be driven by the act of speech synchronisation itself. That is, it is possible that the act of adjusting the timings of one’s speech so as to be in synchrony with an external stimulus could itself induce systematic global changes in F0. There is evidence that speaking conditions associated with increased effort can lead to global changes in F0. For example, when speaking in adverse listening conditions (e.g. background noise or babble), speakers adopt a ‘clear speech’ speaking style that is accompanied by global changes in F0; Hazan and colleagues (52) reported global increases in mean F0 that did not serve to enhance specific phonological contrasts, suggesting they could reflect a by-product of increased speaking effort. In the field of vocal convergence, a study by Kappes, Baumgaertner, Peschke and Ziegler (53) found reduced convergence in F0 in a speech shadowing task (where participants had to repeat non-words with as minimal a delay as possible, even during the ongoing stimulus presentation) compared to a delayed repetition task; they attributed this to an overall tendency to increase F0 due to increased speaking effort in the shadowing task. In a similar way, synchronous speech could have a global effect on F0 due to the increased effort associated with timing the pace of one’s speech with an external stimulus.”*
- The comparison of F0 change between the audio-only and audiovisual condition is motivated by prior literature reporting differences between such conditions for convergence in certain speech tasks (e.g. greater convergence has been reported for an audiovisual compared to an audio-only condition in a live interactive speech task, but not in a speech shadowing task with pre-recorded stimuli; Dias & Rosenblum, 2011; 2016). We have now added more discussion of this literature to motivate inclusion of our audiovisual synchronous speech condition (page 31-32, lines 722-743):
  - *“In addition to comparing visual-only and auditory-only synchronous speech conditions, Experiment 2 further tested a group of participants with a combined audiovisual synchronous speech condition. This allowed us to ask whether being able to see as well as hear the person you are synchronising with would affect convergence to the accompanist voice. Previous research has demonstrated that in some circumstances, visual speech information can enhance vocal convergence to auditory speech over listening to that speech alone. Dias and Rosenblum (54) reported that being able to see as well as hear an interlocutor enhanced convergence during a live interactive search task. Conversely, a follow-up study by the same authors found that visual enhancement of convergence during a non-interactive speech shadowing task was only observed when auditory targets were presented in low-level noise (55). Thus, enhancement of convergence by audiovisual cues may require either live interaction between speakers, or failing that, challenging auditory conditions. We aimed to investigate this in the context of synchronous speech, by comparing F0 changes across audiovisual and audio-only conditions. Since our speech synchronisation task involves neither live interaction between interlocutors, nor challenging auditory conditions, we might predict based on evidence from Dias and Rosenblum that we would not see enhancement of convergence in our audiovisual condition. Alternatively, synchronising one’s speech with a pre-recorded accompanist that can be seen as well as heard may nevertheless increase participants’ perception of a social interaction taking place, resulting in enhanced convergence.”*
- Similar to Experiment 1, we have also now provided a separate ‘Measures and hypotheses’ section for this experiment where we outline the rationale behind our hypotheses and statistical comparisons (pages 43-44, lines 1011-1026).
  - *“Again, our predictions for this experiment stem from our central hypothesis that F0 changes across conditions will be specific to the accompanist voice experienced. For this experiment, we therefore predicted that participants in the audio-only and audiovisual conditions would show significant increases in F0 from solo reading to synchronous speech (to converge to the high F0 accompanist). Conversely, we predicted that participants in the visual-only condition would show no significant change in F0 between these two tasks. This should therefore result in a significant difference in F0 change between the visual-only condition and the two conditions containing audio. Furthermore, to explore whether convergence to an accompanist voice during speech synchronisation is affected by the addition of visual cues, we also compared F0 change in the audio-only and audiovisual conditions. If being able to see as well as hear the accompanist during synchronous speech has a facilitatory effect on convergence, we would expect F0 change in the audiovisual condition to be significantly greater than that in the audio-only condition.”*

12. The large number of exclusions necessary for obtaining a clean sample with 10 participants per condition (13 + 5 + 2 + 1 excluded) needs to be mentioned here. The present conditions for research are highly constrained. Under such conditions, it is more necessary than usual to avoid over- interpreting data. A study like this is valuable, but not if it overreaches. When so many have to be excluded, it seems that it would be wise to state clearly that this is pilot work that can guide future experiments, and no further.

- There has possibly been a slight misunderstanding as to the number of datasets excluded for this experiment; this was 5 + 2 + 5 +1 (13 in total). This is therefore the same number as excluded for Experiment 1 (with the three conditions now combined). For an online study involving audio recording we do not consider this to be an excessive number. The most common reason for exclusion was poor quality audio recordings; we are unsure why this would imply problems with the datasets that did pass our quality checks. However, we of course agree that this study needs replicating and extending in a lab-setting with in-person pairs engaged in synchronous speech; we now emphasise this at the end of our general discussion (page 60, lines 1411-1414):
  - *“It will be important for future research to follow up these preliminary findings with an in-person study of synchronous speech using live interacting pairs, in order to contribute to our understanding of joint speech production.”*

13. Here, the first hypothesis is fine. It emerges naturally from the design of the experiment.

The statistical analysis is overly complex. When you are looking for simple effects, they should be treated as simple effects. T-tests or their multi-condition non-parametric variants would suffice.

- For the group analyses, we have used linear mixed-effect models since we believe this is the most appropriate way to analyse this data. Since this is a repeated-measured design with utterances being nested within participants, these datapoints will tend to correlate with each other. LMMs allow us to explicitly model these random effects, and so are generally recommended as the most appropriate means of analysing data from such a design. We hope that through streamlining the analyses by removing the F0 distance measure, and explaining our hypotheses more thoroughly, the main findings from the study will be easier to interpret.

14. Hypothesis 2 is concerned with magnitudes. See above.

- The F0 distance measure has now been removed so this hypothesis is now concerned with comparing F0 changes between audiovisual and audio-only conditions. We believe that in this case, comparison of magnitudes is warranted since we had a clear hypothesis based on prior literature as to differences between these conditions (since audiovisual cues have been found to provide an enhancing effect on convergence over audio-only cues in certain speech tasks). The reviewer is referred to these papers as a precedent for our analyses:

Dias, J. W., & Rosenblum, L. D. (2011). Visual influences on interactive speech alignment. *Perception*, *40*(12), 1457–1466. <https://doi.org/10.1068/p7071>

Dias, J. W., & Rosenblum, L. D. (2016). Visibility of speech articulation enhances auditory phonetic convergence. *Attention Perception & Psychophysics, 78*(1), 317–333. <https://doi.org/10.3758/s13414-015-0982-6>

15. General discussion. At this point, I think it better not to discuss the argument in detail. My points above mean that the experiment needs to be presented differently. There are simple and interesting effects found.

The complexity and over-elaborate analysis hides the basic findings. The basic findings are to be found in the three tables that report convergence, nothing, divergence:

```
Expt 1
HI       6.    2.    2.
LO       4.    3.    3.
Expt 2
XLO.     4.    3.    3.
Expt 3
VIS.     1.    7.    2.
AUD.     8.    2.    0.
A/V.     8.    0.    2.
```

A few t-tests or non-parametric equivalents would suffice to underscore these findings. They do not speak clearly. There was less convergence than expected.

- Please see our response above regarding the appropriateness of the LMM analysis (point 13). Again, it is our hope that by streamlining the analyses and focusing only on the F0 change measure, the findings now speak much more clearly.

16. The discussion of the significance of these results for specific models is superfluous. It is worth noting in passing that speech science done using neurocentric, individualistic, representational models has ignored or failed to even see joint speech. A dynamical perspective in which coupling and degrees of non-independence can be represented makes it immediately of interest.

- Although we now explicitly acknowledge in the paper that our findings have limited applicability to models of joint action (see pages 59-60, lines 1406-1414, and response to Reviewer point 2), our discussion of models of speech motor control that have been developed through an individualistic perspective is intended to highlight the need for these models to incorporate influences from external voices and speaking partners on speech motor control. There has been increased interest in how principles of control theory that have been used in such models of speech motor control can be applied to the dynamics of speech in conversation (e.g. Gambi & Zhang, 2021, Engineering conversation: Understanding the control requirements of language generation in dialogue. *PsyArXiv*). Our discussion here is aimed at contributing to the movement that requires these theories to consider speech in conversation.

17. So I recommend making this a much smaller paper, with greatly reduced statistical analysis, with clear presentation of what are interesting (if not univocal) results, and keeping an eye towards the design of future experiments under more felicitous conditions.

- We hope that by removing the F0 distance measure and associated analyses, and by combining the original Experiments 1 and 2 into a single Experiment 1, we have now made this a smaller and clearer paper, with easier-to-interpret results. As noted above, we now end our discussion with a call for these preliminary findings to be replicated and extended in a lab-setting with in-person pairs (page 60, lines 1411-1414): *“It will be important for future research to follow up these preliminary findings with an in-person study of synchronous speech using live interacting pairs, in order to contribute to our understanding of joint speech production.”*

**Reviewer 2:**

This is an excellent paper that I am happy to recommend for publication. It provides a thorough and relevant positioning of its original research within the context of previous work on convergence, and in discussion makes connections to possible clinical applications associated with stuttering disorders. Moreover, as a paradigm for effective online data collection, with careful consideration of potential problems, validation methods and norming it has interest beyond its immediate research questions.

*Specific Comments (by line)*

1. 211 mention subject compensation, presumably accomplished through the Gorilla platform

- We have now added this information on page 12 at line 264: “All participants were compensated for their time by payment of £3.75, administered via Prolific.”

2. 455 "Therefore, as predicted, F0 change from baseline was positive in the high condition, but negative in the low condition." While this is clearly correct as visualized in Fig 2, to support this assertion from the LMM results you should also provide the (positive) intercept for the baseline "high" condition.

- We have now added this to our reporting of the model results, so that it is clear that F0 change in the high F0 condition was significantly greater than zero (page 26, lines 596-599):
  - *“This found that F0 change was significantly lower in the low (β = -1.62, t(30.01) = -2.67, p = .012) and extra-low conditions (β = -1.25, t(30.01) = -2.06, p = 0.048) compared to the high condition (the reference condition in the model; Intercept β = 0.99, t(30.14) = 2.32, p = .027).”*

3. 474 "phase" – I had to go back to see what was meant by "phase" here and suggest including something like "phase (solo vs. choral)" to help other readers with that reminder

- Our apologies that this was unclear. We have now removed the F0 distance measure at the request of the Editor and Reviewer 1, and so the variable phase is no longer relevant.

4. 592 In the earlier LMM the "high" level is baseline whereas here "extra-low" is used as baseline; similarly Fig 2 shows a high:low ordering while Fig 5 shows low:high. Suggest reordering levels away from the default alphabetic ordering to make this consistent.

- Experiments 1 and 2 have now been combined into a single analysis and results section, with factor levels reordered so that ‘high’ is the reference condition (with the order high:low:extra-low in Fig.3).

5. 804 "with Tukey['s HSD] adjustment"

- This has now been corrected.

6. 899 "In contrast, our results suggested a tendency for speakers to diverge in F0 from the accompanist during visual choral speech." How can they diverge if they have no access to that cue? A more plausible reason would seem to be something associated with a repetition effect, which you might consider assessing across trials.

- We understand that this wording was somewhat confusing, and have now qualified this as: *“In contrast, our results suggested a tendency for some of our participants to effectively diverge in F0 from the accompanist during visual synchronous speech, due to a slight decrease in F0 (although these changes in F0 were not significant at the group level).*” (page 51, lines 1203-1206). We are presenting our results in contrast to those of Miller et al (2010) who claimed to find convergence to silent visual speech despite no access to auditory cues. We provide a possible interpretation of this decrease in F0 during visual synchronous speech in some of our participants in the previous paragraph (lines 1180-1183): *“This could be driven by a slowing of speech rate and production of flatter intonation patterns during synchronisation (which would affect our median measure of F0 across each sentence).”*

7. 913 This paper is relevant here: Dias, J. W., & Rosenblum, L. D. (2016). Visibility of speech articulation enhances auditory phonetic convergence. Attention, Perception, & Psychophysics, 78(1), 317-333.

- Thank you for bringing this relevant paper to our attention. We now discuss this paper and a related earlier paper from these authors (Dias and Rosenblum, 2011, *Perception*), both when setting up the rationale for Experiment 2 and when interpreting our findings for the comparison between the audiovisual and audio-only conditions. Note that because we have now removed the F0 distance measure, we did not find a significant difference between convergence (F0 change) in these two conditions, in accordance with the findings from Dias & Rosenblum (2016).
- Page 31-32, lines 722-743: *“In addition to comparing visual-only and auditory-only synchronous speech conditions, Experiment 2 further tested a group of participants with a combined audiovisual synchronous speech condition. This allowed us to ask whether being able to see as well as hear the person you are synchronising with would affect convergence to the accompanist voice. Previous research has demonstrated that in some circumstances, visual speech information can enhance vocal convergence to auditory speech over listening to that speech alone. Dias and Rosenblum (54) reported that being able to see as well as hear an interlocutor enhanced convergence during a live interactive search task. Conversely, a follow-up study by the same authors found that visual enhancement of convergence during a non-interactive speech shadowing task was only observed when auditory targets were presented in low-level noise (55). Thus, enhancement of convergence by audiovisual cues may require either live interaction between speakers, or failing that, challenging auditory conditions. We aimed to investigate this in the context of synchronous speech, by comparing F0 changes across audiovisual and audio-only conditions. Since our speech synchronisation task involves neither live interaction between interlocutors, nor challenging auditory conditions, we might predict based on evidence from Dias and Rosenblum that we would not see enhancement of convergence in our audiovisual condition. Alternatively, synchronising one’s speech with a pre-recorded accompanist that can be seen as well as heard may nevertheless increase participants’ perception of a social interaction taking place, resulting in enhanced convergence.”*
- Experiment 2 Discussion, pages 52-53, lines 1220-1240: *“Furthermore, in our study, the addition of visual speech cues to auditory speech information did not result in enhanced convergence relative to an audio-only condition. While some previous work has reported enhanced convergence for audiovisual conditions (54), this effect appears to be specific to tasks involving live interaction between interlocutors. Our findings with a non-interactive task involving pre-recorded stimuli instead accords with findings from Rosenblum and Dias, who similarly found no enhancement of convergence for an audiovisual condition over an audio-only condition in a speech shadowing task with pre-recorded stimuli (55). In that study, an enhancing effect of audiovisual cues on convergence for this non-interactive speech task was only observed when model talker stimuli were presented in low-level noise. Together with our findings, this suggests that in a non-interactive context, visual speech information is only employed to support convergence when auditory speech information is rendered less informative. Interestingly, this appears to be driven by access to speech-relevant articulatory information, and not simply the social relevance of a face stimulus, with Dias and Rosenblum reporting no enhancement of convergence in low-level noise when the mouth area of the model talker was blurred in their video stimuli. Relating this to the Interactive Alignment Model (5,16), access to the articulating mouth may have enabled more accurate or stronger covert imitation of the model talker’s speech via the simulation route, resulting in enhanced priming of representations in the production system and thus increased imitation.”*

8. 917 "perhaps via motor-based speech gesture representations" – the implication here is that F0 can be recovered from facial cues. While there is work suggesting that oral tract constriction gestures can be recovered in this way [e.g. Yehia, H., Rubin, P., & Vatikiotis-Bateson, E. (1998). Quantitative association of vocal-tract and facial behavior. Speech Communication, 26(1-2), 23-43.], I don't see a plausible mechanism for inferring F0.

- We agree that in fact it is unlikely that F0 could be recovered from facial cues; this paragraph has been revised to reflect the updated results and so this sentence has now been removed.

9. 929 "The provision of a dynamic face stimulus in addition to the voice would have increased the salience of the accompanist as a social agent, potentially increasing participants’ motivation for vocal convergence in order to facilitate social bonding with them as an interaction partner." – except that no exchange of social cues takes place with canned stimuli

- The reviewer is correct; we have now adjusted our discussion here to emphasise the difference between synchronisation with a live versus a pre-recorded voice, and how this might affect the impact of visual cues on convergence (page 53, lines 1241-1251):
  - *“Conversely, full visual access to a live interlocutor within an interactive speech task has been reported to enhance convergence compared to a condition where the interlocutor was fully occluded from view (54). It is possible therefore that a similar enhancement of convergence would be seen when engaging in synchronous speech with a live accompanist that could be seen as well as heard. In this context, having visual access to a live accompanist may increase the potential for a two-way exchange of social cues, leading to increased motivation for vocal convergence in order to facilitate social bonding with them as an interaction partner. In this way, whether and how visual cues are used to support vocal convergence appears to differ across different tasks and contexts, particularly with regards to the presence or not of a live interlocutor.”*

10. 976 "recruits additional processes" – this may be so, but should also be discussed in the context of entrainment, which might be providing stability

- We have clarified this phrase slightly, as “recruits additional sensorimotor processes”. However, we are afraid we are unsure as to what the reviewer means by ‘entrainment’ here; does this refer to entrainment to the external rhythm provided by the accompanist, as in traditional interpretations of the fluency enhancing effects of choral/metronome-timed speech, or neural entrainment (i.e. synchronisation between the temporal dynamics of brain responses of the two synchronising partners)? Note that we do already refer to the traditional interpretation of the effects of choral speech in people who stutter in terms of provision of an external rhythm (pages 57-58, lines 1360-1363): *“Traditionally, these have been attributed to the provision of an external rhythm which enables a switch from reliance on a faulty basal ganglia-cortical route for internally timed speech to reliance on an intact cerebellar-cortical route for externally timed speech (63,64).”* If the reviewer could provide more clarification on this point we would be happy to make changes accordingly.

11. 988 "Further research..." It would be interesting and useful for you to speculate on the possible differences between synchronized speech with a single partner vs. true choral speech as part of a synchronized group.

- This point relates to the confusion in our use of terminology as highlighted by Reviewer 1. Our use of the term ‘choral speech’ came from the perspective of the stuttering literature, in which this term refers to synchronised speech between two individuals only. However, we now realise that elsewhere the term ‘choral speech’ is used to refer to synchronised speech across larger groups. To avoid this confusion, we have now adopted the term ‘synchronous speech’ throughout the manuscript (but acknowledge the use of the term ‘choral speech’ in the stuttering literature).

**Reviewer 3:**

The authors report three experiments in which participants spoke in synchrony with recorded speech. The primary issue was whether participants would adapt spoken f0 when synchronizing with a recording of unusually high or unusually low speech. Across experiments, there was a tendency to converge spoken f0 with the recording; however this tendency was stronger for high than for low pitched recordings. In Experiment 3 participants imitated video recordings as well as video+audio for the high-pitched recordings. This experiment demonstrated that the bias for high-pitch convergence was not simply a byproduct of the joint speech task (vocal f0 went down when synchronizing with video only), but also that there was more convergence for video + audio, suggesting that the results may reflect tendencies based on social interactions.
This was an interesting paper and clearly reported. Here are my main concerns.

1. It would be valuable to correlate should know the difference between baseline f0 (from the initial solo reading task) and f0 for synchronization targets with degree of convergence for individuals.

- At the request of the editor and reviewer 1, our F0 distance measure (reflecting the differences between the accompanist F0 and each participant’s baseline F0 and synchronous speech F0) has now been removed from the paper. We now discuss recent evidence suggesting that apparent correlations between distance from the model talker at baseline and magnitude of convergence may in fact be an artefact of the difference-in-distance measure (see page 29, lines 666-678):
  - *“Previous research has suggested that the initial acoustic distance between two speakers can affect the degree of vocal convergence observed, with most studies reporting that convergence is facilitated by a greater distance at baseline (47–49), although the reverse pattern has also been reported (10,50). More recently, it has been argued that the former apparent relationship may in fact be an artefact of the way in which convergence is typically calculated, specifically in relation to the use of a ‘difference-in-distance’ measure (comparing the change in absolute distance between a participant and a model talker from before to after exposure) (51,52). In general, Priva and Sanker (51) argued that measurement of convergence can be unreliable when participants’ baseline values are close to the model talker, as the influence of random variability within an individual is likely to overshadow genuine convergent changes, leading to underestimation of convergence or even apparent divergence.”*
- We have however added some new plots for each experiment showing the difference in semitones between each participant’s F0 during the two tasks and the accompanist F0 (see Figs 1 and 4). These provide a visual illustration of convergence, and enable the reader to clearly see for each participant how far away they were from the accompanist at solo reading, and how close they were able to move during synchronous speech. Given arguments presented by Reviewer 1, we have decided to refrain from conducting any statistical analyses using such a ‘distance’ measure, but we hope that this visual representation will be of use to readers.

2. Sample sizes within each condition were surprisingly small (n=10), and this small sample size was not justified. I know data collection during COVID is difficult, but I think a larger sample of online participants should be possible.

- There are a number of reasons why we had small sample sizes per condition. Firstly, although for many studies the switch from in-person to online data collection allows a much greater number of participants to be tested with increased efficiency, this is not the case for this particular design; data collection was a slow process, requiring quality checking of each individual’s audio recordings that resulted in a high number of exclusions to obtain our final total of 60 participants. Furthermore, although we acknowledge that a simple comparison of two groups of 10 participants as in the original Experiment 1 would warrant caution if presented on its own, this finding of significant convergence was replicated across a number of different conditions. Thus, while our sample sizes in Experiment 1 were also highlighted by Reviewer 2, they point out that “this is mitigated by converging results from the additional experiments.” It should also be acknowledged that these sample sizes are in keeping with those used by other experiments in the field of vocal convergence e.g. Pardo et al., (2012, *Journal of Phonetics*) n = 10, Pardo (2006, *J. Acoust. Soc. Am.*), n = 12, Dias & Rosenblum (2016, *Attention Perception & Psychophysics*) n = 8 per condition.

3. Arguments about applications to stuttering should be limited to discussion because there are no persons who stutter in the experiments. The argument in the discussion could be more clearly described. Why is it beneficial to bring speech motor control away from “internally specified targets and towards external targets”?

- We understand this argument, however the motivation behind and interest in this research topic was driven by the observation of fluency-enhancing effects of synchronous speech in people who stutter, in terms of the insight this provides into how speech motor control may operate very differently in this context; we therefore feel it is appropriate to allude to this in the Introduction.
- We have tried to clarify our argument for why biasing speech motor control away from internally specified targets and towards external targets might be beneficial for people who stutter. The idea that people who stutter have dysfunctional internal models for speech motor control (e.g. unstable feedforward models) has long been hypothesised (e.g. Max, 2004); synchronised speech may decrease the need for reliance on faulty or unstable stored internal speech targets by providing external speech targets to guide speech motor control (page 58, lines 1372-1381):
  - *“Indeed, there is recent evidence for an absence of speech-induced suppression effects in people who stutter (Daliri & Max, 2015; Meekings, Jasmin, Lima, & Scott, 2020), suggesting faulty auditory prediction and internal modelling. Synchronous speech might circumnavigate these dysfunctional processes by biasing speech motor control away from a reliance on faulty or unstable internally specified targets for speech and towards the external targets provided by the accompanist voice. Further research on the effects of synchronous speech in people who stutter, both in terms of potential alignment of their speech productions with the accompanist and the nature of neural mechanisms of prediction during synchronous speech, would provide insights into these ideas.”*

4. The shift of f0 was more extreme for low than for high targets. Do we know that both targets sounded similarly natural? Could lower tendency to converge to low targets reflect the greater loss of naturalness from the more extreme shift?

- The natural F0 of the female voice which provided the voice recordings for the accompanist stimuli was already quite high, and so a smaller upwards F0 shift was required to obtain stimuli with an unusually high average F0. It should be noted that the debrief questionnaire at the end of the experiment asked all participants to report if they noticed anything about the other voice, and none reported that it sounded unnatural. Therefore we have no reason to believe that stimuli in the low condition sounded less natural than in the high condition.

Minor comments

5. Lines 413-417: This is a somewhat questionable use of t-test as it assumes independent sampling across trials for a participant.

- At the suggestion of the Editor and Reviewer 1, these t-tests now test whether the change in F0 in semitones from solo reading to synchronous speech is significantly different from zero (rather than testing whether the change in distance from the accompanist between solo reading and synchronous speech was significantly different from zero as in the original paper). A possible alternative would be to use a paired-samples t-test to test for a significant difference between the participant’s F0 values at solo reading and those at synchronous speech. However, since we have been advised to use a semitone scale for examining changes in F0, we are constrained to having to first calculate the F0 change in semitones, and then using a one-sample t-test. Note that this approach also has precedence in the altered auditory feedback literature, where one-sample t-tests on difference scores are regularly used to determine if individual participants showed significant adaptation or not (e.g. Lametti, Smith, Watkins & Shiller, 2018; Lametti, Nasir & Ostry, 2012).

6. Lines 430-432: Technically correct, but the lowest participant in the low-voice group is for all practical purposes identical in pitch to the low voiced target.

- The reviewer is quite right, and this is precisely why we decided to design and test an additional group of participants with an accompanist voice with an even lower F0 in our extra-low condition. Since the original Experiments 1 and 2 have now been combined into a single analysis at the request of the Editor and Reviewer 1, we now discuss how the distribution of F0 values observed in our sample during the solo reading baseline task motivated the design of the additional extra-low F0 condition (pages 24-25, lines 561-568):
  - *“From these descriptives, it can be seen that the range of F0 values measured during solo reading in the low F0 group overlaps with the average F0 of the accompanist voice to which they were subsequently exposed in the synchronous speech task (170Hz). Since it was our aim to use accompanist voices whose average F0 was far away from that of our sample at solo reading, this motivated the design of our additional extra-low F0 accompanist condition. In this condition, the accompanist voice’s average median F0 (140Hz) was substantially lower than the range observed in our initial sample.”*

7. Line 455: Why are there 20 degrees of freedom for t. With n=10 per sample, a t-test on independent samples should be df=18 I would think.

- The reported statistics do not reflect a t-test on independent samples, but reflect the statistics for the fixed effects of the linear mixed effect model provided by the lmerTest package in R. This implements Satterthwaite’s method of approximating degrees of freedom. As explained in Kuznetsova, Brockhoff and Christensen (2017, *Journal of Statistical Software*), this is required for LMMs because “generally the exact null distributions for the parameter estimates and test statistics are unknown. So the only way to judge about the significance of the effects is by some sort of approximation and/or simulation based approach.” Please see the below screenshot of the output of the summary of the model fitted using lmerTest:

```
Fixed effects:
                   Estimate Std. Error      df t value Pr(>|t|)
(Intercept)          0.9987     0.4301 30.1447   2.322   0.0272 *
ConditionLow        -1.6230     0.6076 30.0120  -2.671   0.0121 *
ConditionExtra_low  -1.2540     0.6076 30.0131  -2.064   0.0478 *
---
Signif. codes:  0 ‘***’ 0.001 ‘**’ 0.01 ‘*’ 0.05 ‘.’ 0.1 ‘ ’ 1
```

8. Figure 1: Curious that there does not seem to be a strong trend across trials. Why?

- This is a very interesting question that relates to our discussion as to whether convergence responses can be viewed within the same framework as sensorimotor adaptation during altered auditory feedback. Whereas the adaptation response typically builds up gradually across trials, the reviewer rightly points out that for Experiment 1, change in F0 does not appear to show a strong increasing trend across trials. This is consistent with previous studies of F0 convergence; for example, a study by Aubanel and Nguyen (2020) looking at convergence in F0 in a shared (not synchronous) reading task found that the maximal level of convergence was achieved at the beginning of the task, rather than convergence ramping up across turns. Conversely, in our Experiment 2 (previously Experiment 3), there is a suggestion of a gradual increase in convergence across trials, particularly in the audiovisual condition which shows a strong upward trend. It is possible that this trend is seen more strongly in this experiment due to the reduced stimulus set (only 3 sentences repeated across trials instead of using trial-unique sentences in experiment 1). We have now added a discussion of these points to the general discussion (page 56-57, lines 1326-1346):
- *“One interesting comparison to make between the sensorimotor adaptation responses seen in altered auditory feedback studies and vocal convergence responses concerns their time-scale. Adaptation responses to altered self-voice feedback typically ramp up over a series of trials (60–62). Conversely, in our first experiment, F0 change did not appear to linearly increase across the task (see Fig 2). This is consistent with some previous studies of convergence; for example, a study of F0 convergence during a shared reading task by Aubanel and Nguyen found that the maximal level of convergence was achieved at the beginning of the task, then remaining relatively stable across the interaction (10). However, in our second experiment, convergence did appear to show an upward trend across trials, particularly in the audiovisual condition (see Fig 5). One possible reason for this difference in the pattern of convergence between experiments could lie in the use of a restricted stimulus set in Experiment 2 (only three sentences were repeated across trials, in contrast to the trial-unique sentences used in experiment 1). This is perhaps more similar to the design of altered auditory feedback experiments in which the same small set of stimuli is typically repeated across multiple blocks. Some authors have argued that convergence in different cues can take on multiple different temporal patterns, either continuously increasing across an interaction or dynamically fluctuating according to a speaker’s motivational state and intentions (63). It will be useful for future studies of vocal convergence to consider more closely the trends in convergence responses across time, and what factors might affect the temporal dynamics of the response.”*

9. Figure 3 (and similar figures): State what acronyms mean in the caption.

- We have now revised all figure captions to ensure that all variables presented on graphs are clearly explained. Since we no longer present the F0 distance measure, the acronyms CS and SR are no longer used on graphs.

10. Table 2: Not sure necessary. Also not clear why 20.4 df for each mean (shouldn’t this be 9 per condition?)

- At the request of the Editor and Reviewer 1, this analysis of the F0 distance measure has now been removed.

11. Lines 522-523: What cultural differences predict lower f0 for British-English female speech in contrast to American English? Why is this not an artifact of the small sample?

- Since we have now combined Experiments 1 and 2 into a joint analysis, we no longer include this discussion of potential differences between our British-English sample and the UCLA sample. The reviewer is quite right that this may simply be an artefact of the small sample. For the purposes of our study, we simply wanted a condition in which the average F0 of the accompanist voice was far away from that of the participants in the sample exposed to that voice; this was then achieved in the extra-low condition.
