# Supplementary material for: Convergence in voice fundamental frequency during synchronous speech
Source: PLoS One. 2021 Oct 21;16(10):e0258747. doi: 10.1371/journal.pone.0258747 (PMC8530294; doi:10.1371/journal.pone.0258747)
Supplement: Attachment — Submitted filename: Response to Reviewers.docx [file pone.0258747.s006.docx]

**Response to Reviewers**

**Second Resubmission**

We would like to thank the editor and all three reviewers for their helpful comments throughout peer review of this manuscript, and are pleased that they were happy with our first revision. We have now fully addressed all outstanding points raised by the editor in this second revision, and hope that the paper will now be acceptable for publication. Please note that page and line numbers given below refer to the manuscript with tracked changes.

**Editor’s comments:**

1. In the General Discussion, provide an acknowledgement of the relatively small sample size and an explanation of how this compares with sample sizes of similar published work. Then please include a statement addressing generalizability given your sample size. The aim of this is to give readers who are not working in this area a sense of where the study sits in this respect.

- We have added the following lines to the limitations section within our General Discussion (see page 42, lines 989-995):
- *“Some limitations of the current study must be acknowledged when interpreting the results. Firstly, relatively small sample sizes were used for our conditions (n = 10 per condition). Although these are in keeping with sample sizes used by others in the field of vocal convergence (e.g. 57,73,74), our findings should therefore be viewed as preliminary and in need of further replication. It appears promising, however, that we were able to replicate significant convergence across multiple different synchronous speech conditions in two separate experiments.”*

2. Reviewer 1 argues that in their view ‘synchronized’ speech should be reserved for laboratory speech. While I am not in a position to judge whether such use of terminology is warranted and aligns with a common view, please include a sentence clarifying whether you intend synchronous speech to be reserved for laboratory studies or not, and how this aligns with the literature. Again, my aim is to achieve maximal terminological clarity to allow readers from different areas and theoretical persuasions to fully benefit from reading your work.

- We have now tried to clarify our definition of the term ‘synchronous speech’ in the introduction. In the first paragraph we start by using the terms *“synchronised vocal behaviours”* and *“joint speech”* to refer more generally to cases where speakers produce speech in unison in everyday life (in line with Reviewer 1); in the second paragraph we then introduce and define the term *“synchronous speech”* to refer specifically to the task of reading a prepared text in synchrony with another speaker, while acknowledging other terms used in the literature (see page 3, lines 62-65): *“Strikingly, reading a prepared text in synchrony with another speaker (here termed synchronous speech; also referred to as “choral speech” or “choral reading” in this literature) can temporarily reduce…”*. Thereafter we use this consistent term to refer to this task (whether it is used in a laboratory setting or as a temporary fluency enhancer with people who stutter). We hope that this consistency in the use of a single term will provide greater clarity for readers from outside the field, while the acknowledgement of other terms used in different fields for the same task at this early point in the introduction will allow maximal terminological clarity across readers working within these different areas.

3. Reviewer 2 provided a clarification of their earlier statement about entrainment – please check how your interpretation aligns with their conceptualization of entrainment and perhaps add a mention of the stated interpretation in your Discussion.

- With this additional clarification from Reviewer 2, we have now added a new short paragraph to the General Discussion outlining this interpretation of convergence effects during synchronous speech in terms of entrainment, and how this might provide stability for people who stutter (see pages 41-42, lines 974-988):
- *“Alternatively, the beneficial effects of synchronous speech in people who stutter may relate to engagement of an entrainment process (73) that drives changes in the acoustics of speech productions. This interpretation of vocal convergence based on dynamical systems theory (74) was put forward by Pardo (75), who suggested that a pair of interacting talkers can be viewed as an informationally coupled dynamical system. Within such a system, a ‘magnet effect’ occurs in which externally derived information (i.e. the other speaker’s voice) acts as a forcing function on internal dynamics; over time, the more dominant talker pulls the less dominant talker into coordination so that their dynamics (i.e. vocal acoustics) more closely resemble each other, resulting in relative (but not absolute) coordination. This kind of entrainment process may provide stability or an alternative route for speech motor control in people who stutter. Further research on the effects of synchronous speech in people who stutter, both in terms of potential convergence of their speech productions with the accompanist and the nature of neural mechanisms of prediction during synchronous speech, would provide insights into these ideas.”*

4. Reviewer 3 points out interesting links to the literature on musical synchronization – I leave it up to you whether you see value in pointing out or even elaborating briefly on these links in the general Discussion.

- We are grateful to Reviewer 3 for pointing us to this literature, which indeed has some interesting parallels to the current work. On this occasion however, we feel that there is not a natural point in the General Discussion at which to allude to this work.
